# The Survival and Prognostic Factors of Supratentorial Cortical Ependymomas: A Retrospective Cohort Study and Literature-Based Analysis

**DOI:** 10.3389/fonc.2020.01585

**Published:** 2020-08-21

**Authors:** Qiguang Wang, Jian Cheng, Jiuhong Li, Si Zhang, Wenke Liu, Yan Ju, Xuhui Hui

**Affiliations:** Department of Neurosurgery, West China Hospital, Sichuan University, Chengdu, China

**Keywords:** cortical ependymomas, clinical features, treatment, outcome, RELA fusion

## Abstract

**Aim:**

Survival rates and prognostic factors of cortical ependymomas (CEs) remain elusive. This study aimed to perform a comprehensive analysis of prognostic factors, treatment, and outcomes for patients with CEs based on institutional and literature case series.

**Materials and Methods:**

Thirty patients with CEs from our department were included in this study. Furthermore, a systemic review of the literature yielded an additional 106 patients with CEs. Clinical data including patient age, sex, symptoms, tumor location, World Health Organization (WHO) grade, extent of surgery, radiation, recurrence, and survival were recorded and statistically analyzed.

**Results:**

From January 2009 to October 2019, 30 (4.2%) cases were diagnosed as CEs in our department. These series consisted of 19 males and 11 females, 10 continuous patients after 2017 screened for C11orf95-RELA fusion, and 9 patients (90%) were RELA fusion positive. During the follow-up period, nine (30%) patients depicted tumor recurrence or progression; four (13.3%) patients died of tumor progression. The literature review yielded 106 CE cases, with additional 30 cases of our own collected for further analysis. Of these 136 cases, the frontal lobe (40%) was the most common location, and the average age was 22.6 ± 17.6 years. Anaplastic histology/WHO grade III tumors were identified in 68 (50%) patients. Statistically analysis demonstrated that extent of surgery and WHO tumor grade were significant prognostic factors in Kaplan–Meier log-rank testing and Cox proportional hazards models. Gross total resection (GTR) predicted longer progression-free survival (PFS) [*P* = 0.013, hazard ratio (HR) = 3.012, 95% confidence interval (CI) = 1.257–7.213] and overall survival (OS) (*P* = 0.003, HR = 5.322, 95% CI = 1.751–16.178). WHO grade III tumors had worse PFS (*P* = 0.002, HR = 5.17, 95% CI = 1.804–14.816) and OS (*P* = 0.025, HR = 5.640, 95% CI = 1.248–25.495).

**Conclusion:**

CEs accounted for only 3.5 to 5.7% of ependymomas, with seizures the most common symptom and the frontal lobe the most frequent location. CEs may have higher rate of RELA fusions, but generally favorable prognosis. The extent of surgery and WHO tumor grade were significant prognostic factors for PFS and OS in multivariate analysis. GTTR or WHO grade II tumors had better overall outcome in patients with CEs.

## Introduction

Ependymomas are rare central nervous system tumors and account for 2 to 9% of all neuroepithelial tumors (1, 2). They predominantly involve the fourth ventricle and spinal cord (3, 4). Supratentorial ependymomas (STEs) approximately account for 30 to 50% of intracranial ependymal tumors (5). Cortical ependymomas (CEs) are such STEs that selectively involve cerebral cortex and have no relationship with the ventricular system (6–8).

Several previous studies have indicated that CEs are not a topographic description, but a new distinct subtype of STEs and should be classified separately from other types of ependymomas (5, 9–11). However, because of the rarity of CEs, most publications detail only simply constructed case reports or retrospective studies with small sample size (<10), the clinical features and outcome of CEs are still not elucidated. Hence, to ensure the appropriately treatments are performed, it is necessary to further identify clinical features and prognostic factors of CEs.

A large-scale clinical investigation of CEs is lacking in the literature. In the present study, we retrospectively reviewed our institutional series and summarized the literature data to more accurately determine the optimal treatment and prognosis of CEs.

## Materials and Methods

### Patient Population and Data Collection

Between January 2009 and October 2019, 709 cases of ependymomas underwent surgical resection at the Department of Neurosurgery, West China Hospital of Sichuan University. Seventy-one (10%) cases were found in supratentorial extraventricular location, and 30 (4.2%) cases were diagnosed as CEs. Clinical data for patients were retrospectively sourced after having gained approvals from the Institutional Review Board of West China Hospital. Data on patient age, sex, clinical and radiological features, World Health Organization (WHO) tumor grade, treatment, and outcome data were retrospectively collected. Interphase fluorescence *in situ* hybridization was undertaken to screen for C11orf95-RELA fusion for patients with ependymoma in our series since 2017. Radiological data included tumor size, location, enhancement patterns, and tumor texture (cystic, solid, or cystic-solid). Data for treatment included extent of tumor resection [gross total resection (GTR), subtotal resection (STR), or partial resection (PTR)], postoperative radiation, and chemotherapy. The extent of surgical resection was determined by reviewing the postoperative magnetic resonance imaging (MRI) scans. Based on volumetric analysis using postoperative MRI, GTR was defined as complete resection of the tumor; subtotal resection was defined as any incomplete resection with less than 10% of tumor remnant; PTR was considered if the remnant tumor was larger than 10% of the initial tumor in volume. All patients received regular follow-ups, and gadolinium (Gd)–enhanced intracranial MRIs were conducted to assess the tumor recurrence or progression. The outcome data included the follow-up time, the time of recurrence, the progression-free survival (PFS) time, and overall survival (OS) time. PFS time was defined as the interval from the date of surgery to the time of tumor recurrence or progression; OS time was defined as the interval from the time of surgery to death.

### Systematic Analysis

We collected data of patients with CEs according to the PRISMA (Preferred Reporting Items for Systematic Reviews and Meta-Analyses) guidelines (12). We searched PubMed and collected the literature data up to September 2019, using the keyword “cortical ependymoma.” All prior studies (case reports and case series) with disaggregated clinical information were included in this study. We next extracted clinical information for each patient regarding patient gender, age, clinical features (seizures or non-seizures), radiological manifestations (tumor location and tumor texture), WHO tumor grade, treatment (extent of surgery resection, postoperative radiation, and chemotherapy), and follow-up data (follow-up time, time of tumor recurrence, PFS, and OS). Subjects lacking clinical data were excluded from statistical analyses. In addition, we comparatively assessed the clinical characteristics between our local cohort and literature cohort.

### Statistical Analysis

Patient age, PFS, and OS were analyzed as continuous variables. Patient age (>16 or ≤16 years), gender, tumor location (single lobe or multiple lopes), patient symptoms (seizures or non-seizures), tumor texture (solid, cystic, or solid–cystic), WHO grade, extent of surgery, and postoperative radiation were analyzed as categorical variables. Survival outcomes were assessed using PFS and OS. Univariate survival analysis was constructed both for PFS and OS, using the Kaplan–Meier method and log-rank tests to identify the prognostic factor. Multivariate survival analysis using Cox’s regression model was performed for variables that were significant on the univariate analysis (*P* < 0.05). All statistical analyses were performed with SPSS version 22 software (version 22.0; IBM, Chicago, IL, United States). *P* < 0.05 was set as the level of statistical significance.

## Results

### Patient and Tumor Characteristics

**FIGURE 1:**
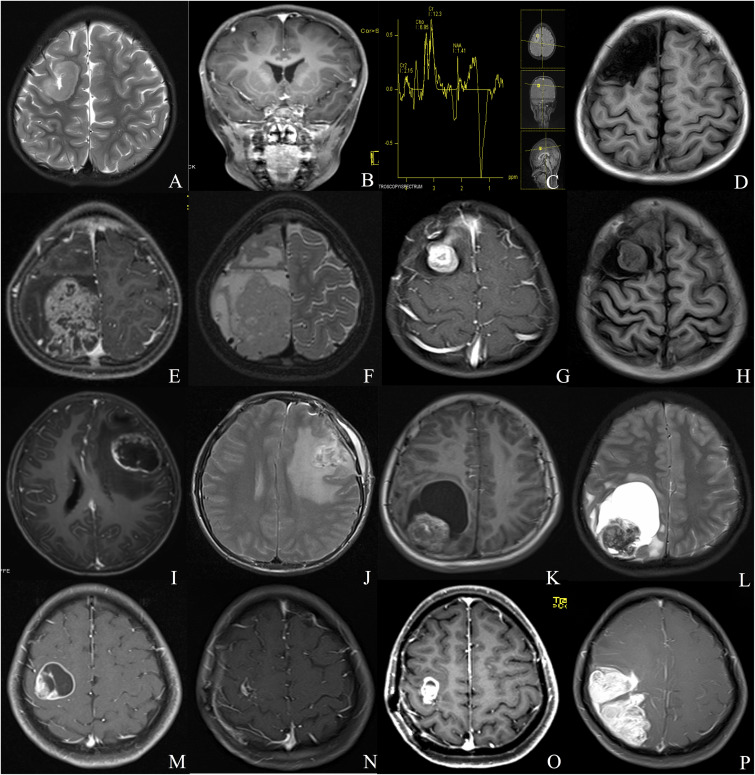
Different radiologic characteristics of CEs. Case 1 **(A–D)** The tumor was found in the right frontal cortex **(A)** and showed no obvious enhancement **(B)**; MRS depicted increased choline and decreased *N*-acetyl-aspartate **(C)**; postoperative MRI scan showed total resection of the tumor **(D)**. Case 2 **(E,F)** A solid tumor with heterogeneous enhancement was depicted in the right parietal lobe. Case 3 **(G,H)** The tumor showed homogeneous enhanced solid appearance on MRI **(G,H)**. Case 4 **(I,J)** The tumor depicted a rim-enhanced cystic lesion with obvious peritumoral edema. Case 5 **(K,L)** The tumor demonstrated a solid mural with peritumoral cyst. Case 6 **(M–P)** The lesion showed rim-enhanced cystic lesion, without peritumoral edema **(M)**; subtotal resection was applied due to the central region location of the tumor **(N)**; the tumor recurred 1 year after the surgery **(O)**, and the lesion continued to grow during the follow-up **(P)**.

Our institutional series included 19 patients were male and 11 were female (Table 1). The two most common locations were temporal (26.7%) and frontal (23.3%) lobes. Nine (30%) patients experienced seizures preoperatively, and the other common symptoms included headache (15, 50%) and weakness (4, 13.3%). Radiologically, 13 (43.3%) patients demonstrated a solid appearance; 11 (36.7%) patients showed cystic manifestation, and the other six (20%) patients revealed concomitant solid and cystic appearance. Twenty-seven (90%) patients exhibited different types of enhancement, including mild to moderate heterogeneous enhancement and rim enhancement; three patients depicted no obvious enhancement (Figure 1). Magnetic resonance spectroscopy (MRS) of CEs frequently characterized increased choline and decreased *N*-acetyl-aspartate (Figure 1). Seventeen tumors were diagnosed as grade II (ependymoma), whereas the remaining 13 tumors were diagnosed as WHO grade III (anaplastic ependymoma). Ten patients after 2017 screened for C11orf95-RELA fusion based on the recent 2016 WHO classification system, and 9 of the 10 patients (90%) were RELA fusion positive. As for the treatments, 20 patients (66.7%) underwent GTR, and 10 patients (33.3%) received STR. Furthermore, 14 patients received postoperative local irradiation with a median dose of 50 Gy. At average follow-up of 33.0 ± 20.2 months, nine (30%) patients showed tumor recurrence or progression; four (13.3%) patients died of tumor progression.

**TABLE 1:** Overview of patient data of our institutional series.

Patient number	Age (years)	Sex	Location	Symptoms	Tumor texture	WHO grade	RELA fusion	Treatment	Follow-up (months)	Recurrence	Outcome
1	26	Female	Rt frontal	Seizures	Cystic	III	NA	STR	43	Yes	Dead
2	48	Male	Lt temporal	Headache, dizziness	Cystic with mural nodule	II	NA	STR + RT	94	Yes	Alive
3	50	Male	Rt frontal	Headache	Solid	II	Yes	GTR	24	No	Alive
4	5	Female	Rt frontal	Seizures	Solid	II	NA	GTR	24	No	Alive
5	5	Male	Lt frontotemporal	Headache, vomiting	Cystic with mural nodule	III	NA	GTR + RT	50	No	Alive
6	54	Female	Rt temporal	Dizziness	Cystic	III	No	STR + RT	26	Yes	Dead
7	8	Female	Rt temporal-occipital	Headache	Solid-Cystic	III	NA	STR + RT	21	Yes	Alive
8	37	Male	Rt temporal	Headache	Solid	II	NA	STR + RT	36	Yes	Alive
9	22	Female	Rt temporal	Headache, vomiting	Solid	II	NA	STR + RT	29	Yes	Dead
10	58	Male	Rt temporal	Seizures	Cystic with mural nodule	II	NA	GTR	60	No	Alive
11	17	Male	Rt frontotemporal	Headache, vomiting	Cystic	II	NA	STR + RT	48	No	Alive
12	2	Female	Rt frontal	Headache	Solid-Cystic	III	NA	GTR + RT	36	No	Alive
13	4	Male	Rt occipital	Headache, vomiting	Cystic with mural nodule	II	NA	GTR	36	No	Alive
14	6	Female	Rt frontoparietal	Left side weakness	Cystic	II	NA	GTR	30	No	Alive
15	5	Male	Rt frontal	Seizures	Solid	II	Yes	GTR	29	No	Alive
16	7	Male	Rt occipital	Headache	Solid	II	NA	GTR	9	No	Alive
17	11	Female	Rt temporal	Headache	Solid-Cystic	II	NA	GTR	36	No	Alive
18	6	Female	Lt temporal-occipital	Seizures	Solid	II	Yes	GTR	18	No	Alive
19	11	Male	Lt temporal	Seizures	Solid	III	Yes	GTR + RT	18	No	Alive
20	13	Female	Rt parietal	Headache, vomiting	Cystic	III	NA	STR + RT	48	No	Alive
21	2	Male	Lt frontoparietal	Right side weakness	Solid-Cystic	III	NA	STR	24	Yes	Dead
22	19	Female	Rt temporal-occipital	Seizures	Cystic	II	NA	GTR	27	No	Alive
23	4	Male	Rt parietal	Headache, vomiting	Solid	III	Yes	GTR + RT	36	No	Alive
24	17	Male	Rt occipital-temporal	Seizures	Cystic with mural nodule	II	Yes	GTR	8	No	Alive
25	22	Male	Left parietal	Seizures	Solid	III	Yes	GTR + RT	6	No	Alive
26	4	Male	Rt temporal	Headache, vomiting	Solid	II	NA	GTR	65	No	Alive
27	5	Male	Rt frontal	Headache, vomiting	Solid	III	NA	GTR + RT	72	Yes	Alive
28	14	Male	Rt frontotemporal	Right side weakness	Solid-Cystic	II	NA	GTR	24	No	Alive
29	11	Male	Rt frontoparietal	Left side weakness	Solid	III	Yes	STR + RT	7	Yes	Alive
30	0.75	Male	Rt frontal	Vomiting	Solid-Cystic	III	Yes	GTR	5	No	Alive

*Rt, right; Lt, left; GTR, gross total resection; NA, not application; STR, subtotal resection; RT, radiotherapy.*

A total of 136 patients, including 106 cases from the literature (Table 2) and 30 from our department, were collected for further statistical analysis. The average age of the whole patient group was 22.6 ± 17.6 years, with 76 (55.9%) patients male and 60 (44.1%) female. The frontal lobe (46, 40%) was the most common location, followed by parietal (24, 20.3%) lobes, temporal lobes (18,15.3%), and occipital lobes (2.5%). The symptom of seizures documented in 61 (45.2%) of patients and other common symptoms included headache (54, 39.7%), weakness (13, 9.6%), and so on. Most of the tumors showed heterogeneous enhancement on Gd-enhanced MRI. Forty-three cases (37.1%) exhibited solid appearances with mild to moderate heterogeneous enhancements, 46 (39.7%) patients showed cystic appearance, and most tumors depicted a large enhanced cyst with enhanced mural nodule; the remaining 27 (23.2%) patients depicted concomitant solid and cystic appearance. Based on the WHO classification, 68 (50%) tumors were diagnosed as grade II (ependymoma) and 68 (50%) tumors were diagnosed as WHO grade III (anaplastic ependymoma).

**TABLE 2:** List of literature papers included in our analysis (*n* = 35).

Papers included	No. of patients included	Papers included	No. of patients included
(38)	1	(39)	1
(40)	1	(41)	1
(42)	1	(43)	1
(44)	1	(45)	1
(7)	1	(46)	1
(5)	3	(47)	1
(48)	1	(49)	1
(50)	1	(8)	1
(51)	2	(20)	11
(52)	1	(53)	1
(54)	1	(55)	1
(56)	3	(57)	1
(58)	1	(59)	1
(60)	1	(2)	13
(61)	9	(62)	1
(63)	1	(64)	18
(65)	1	(13)	13
(11)	8		

### Treatments

All patients in the present study received surgical resection. One hundred eleven (81.6%) patients underwent GTR, and 25 (18.4%) patients underwent STR of the tumors (Table 3). Postoperatively, 62 (45.6%) patients received irradiation. Only five patients underwent postoperative chemotherapy, and this factor was not included in the univariate and multivariate analyses.

**TABLE 3:** Baseline characteristics of CEs, and a comparisonxs between literature and local cohorts.

Variable	All	Local cohort (*n* = 30)	Literature cohort (*n* = 106)	*P-*value
**Sex**				
Male	76(55.9%)	19(63.3%)	57(53.8%)	0.352
Female	60(44.1%)	11(36.7%)	49(46.2%)	
**Location**				
Single lobe	90(76.3%)	20(66.7%)	70(79.5%)	0.152
Multiple lobes	28(23.7%)	10(33.3%)	18(20.5%)	
**Symptoms**				
Seizures	61(45.2%)	9(30.0%)	52(49.5%)	0.058
Others	74(54.8%)	21(70.0%)	53(50.5%)	
**Texture**				
Solid	43(37.1%)	12(41.4%)	31(35.6%)	0.846
Cystic	46(39.7%)	11(37.9%)	35(40.2%)	
Solid-Cystic	27(23.2%)	6(20.7%)	21(24.1%)	
**WHO Grade**				
II	68(50%)	17(56.7%)	51(48.1%)	0.408
III	68(50%)	13(43.3%)	55(51.9%)	
**Surgery resection**				
Subtotal resection	25(18.4%)	10(33.3%)	15(14.2%)	0.017
Gross total resection	111(81.6%)	20(66.7%)	91(85.8%)	
**Irradiation**				
Yes	62(45.6%)	14(46.7%)	48(45.3%)	0.893
No	74(54.4%)	16(53.3%)	58(54.7%)	
**Recurrence**				
Yes	37(28.5%)	9(30%)	28(28%)	0.831
No	93(71.5%)	21(70%)	72(72%)	
**Outcome**				
Alive	115(89.8%)	26(86.7%)	89(90.8%)	0.51
Dead	13(10.2%)	4(13.3%)	9(9.2%)	

### Outcomes

After average follow-up of 44.3 ± 46.5 months (range, 2–264 months), tumor progression or recurrence occurred in 37 (28.5%) cases, and 13 (10.2%) patients died at the end of follow-up.

### Univariate and Multivariate Analyses of Prognostic Factors for PFS

**FIGURE 2:**
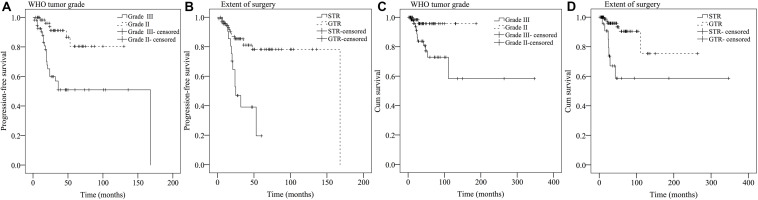
Kaplan–Meier survival curves of prognostic factors significant in multivariate analysis for PFS **(A,B)** and OS **(C,D)**.

Results from univariate analysis for PFS are demonstrated in Table 4. Tumor location (*P* = 0.027), patient symptoms (*P* = 0.01), WHO tumor grade (*P* < 0.001), extent of surgery (*P* < 0.001), and postoperative irradiation (*P* = 0.014) were significant prognostic factors for PFS. Multivariate analysis indicated WHO tumor grade and extent of surgery were significant prognostic factors (Table 5). Patients with WHO grade II CEs had longer PFS than those with WHO grade III tumors [*P* = 0.002, hazard ratio (HR) = 1.804–14.816]. Patients who underwent GTR had longer PFS than those who did not (*P* = 0.013, HR = 1.257–7.213). Kaplan–Meier survival curves for variables significant in multivariate analysis for PFS are shown in Figures 2A,B.

**TABLE 4:** Univariate analysis of prognostic factors for PFS and OS.

Factor	Categories	PFS		OS	
		Numbers	*P*-value	Numbers	*P*-value
Patient age	>16 years	64	0.962	68	0.312
	≤16 years	56		59	
Sex	Male	68	0.579	73	0.813
	Female	52		54	
Location	Single lobe	82	0.027*	87	0.54
	Multiple lobes	24		24	
Symptoms	Seizures	57	0.010*	60	0.057
	Others	62		66	
Texture	Solid	41	0.661	42	0.83
	Cystic	39		43	
	Solid-Cystic	24		24	
WHO Grade	II	65	< 0.001*	65	0.011*
	III	55		62	
Surgery resection	Subtotal resection	23	< 0.001*	24	0.001*
	Gross total resection	97		103	
Irradiation	Yes	54	0.014*	58	0.743
	No	66		69	

*Statistically significant.

**TABLE 5:** Multivariate analysis related with PFS and OS.

Clinical factors	Hazard ratio	*P-*value	95% CI
**Multivariate analysis related with PFS**			
Location	0.478	0.120	0.188–1.213
Symptoms	0.580	0.307	0.204–1.650
Irradiation	0.921	0.866	0.353–2.398
WHO Grade			
Grade II	5.170	0.002*	1.804–14.816
Surgery resection			
GTR	3.012	0.013*	1.257–7.213
**Multivariate analysis related with OS**			
WHO Grade			
Grade II	5.640	0.025*	1.248–25.495
Surgery resection			
GTR	5.322	0.003*	1.751–16.178

*Statistically significant.

### Univariate and Multivariate Analyses of Prognostic Factors for OS

Findings for univariate and multivariate analyses of prognostic factors for OS are presented in Tables 4, 5. Only preoperative WHO tumor grade (*P* = 0.011) and the extent of surgical resection (*P* = 0.001) were observed to have statistically significant difference for OS. Patients with WHO grade II CEs had longer OS than patients with WHO grade III tumors (*P* = 0.025, HR = 1.248–25.495). Patients who underwent GTR had longer OS than those who did not (*P* = 0.003, HR = 1.751–16.178). Kaplan–Meier survival curves for variables significant in multivariate analysis for OS are shown in Figures 2C,D.

### Cohort Difference

Compared with the data from the literature cohort, the local cohort had no significant differences with respect to patient gender (*P* = 0.352), tumor texture (*P* = 0.846), and WHO tumor grade (*P* = 0.408). The tumors frequently involved a single lobe in both the present cohort and literature series (*P* = 0.152). The rate of GTR for tumors in the present series was lower than that in the literature (*P* = 0.017). Compared with the literature studies with CEs, our patients showed no significant difference in PFS and OS. The recurrence rate and mortality for CEs in our series were 30 and 13.3% compared with 28 and 9.2% in the literature series (Table 3).

## Discussion

Because of the rarity of CEs, single-center based studies are underpowered to generate any statistical significance. Hence, retrospectively collecting both the institutional and literature data can help to overcome the small sample size. Our study, consisting of both institutional series and literature data, represents the largest clinical investigation of CEs completed to date.

Various prior studies considered that CEs were not only a topographic description, but may be a “new” WHO entity. Roncaroli et al. (5) hold that CEs should be separate from other ependymomas and postulated CEs had indolent nature with favorable prognosis after surgery resection. Matsumoto et al. (11) indicated that CEs had comparatively favorable outcomes despite the high rates of C11orf95-RELA gene fusion, and they hold CEs should be classified as a new distinct subtype of STEs. Therefore, it is essential to further illuminate the epidemiology, clinical characteristics, treatment, and outcome for patients with CEs.

The exact incidence of CEs remained controversial. In the past 11 years, 709 central nervous system ependymomas underwent surgical resection in our department. Of these, only 30 (4.2%) cases were identified as cortical location. In supporting of our findings, Wang et al. (2) found only 3.5% of ependymomas were located in the cortex. Likewise, Van Gompel et al. (9) depicted only 4% of ependymomas were diagnosed as CEs. Sun et al. (13) stated CEs accounted for 5.7% of ependymomas in their institutional series. Hence, CEs represented a rare subgroup of ependymomas and accounted for only 3.5 to 5.7% of intracranial ependymomas.

Our local cohort depicted that CEs had a preference for male and always located in single lobe, with temporal lobe as the most common location (26.7%). The most common symptoms in the present series were those related to increased intracranial pressure such as headache or vomiting. Our institutional series showed higher rate of WHO grade II pathology in CEs. The subtotal resection rate (33.3%) in our local series was higher than that in the literature series (14.2%). The high rate of STR was likely attributed to the tumors always being close to or invading the crucial structures such as cerebral central region, the basal ganglia, thalamus, and so on; in order to protect the important functional brain areas in some cases, there may be some residual tumors in some cases.

RELA fusion-positive ependymoma has been considered as a novel entity according to the 2016 WHO classification of CNS tumors (14). Previous studies depicted that incidence of C11orf95-RELA fusions in STE ranging from 65.1 to 70% (15–17). However, the incidence of C11orf95-RELA fusions in CEs has rarely been investigated before. Our series depicted that CEs had a higher rate of C11orf95-RELA fusions, with 9 of 10 (90%) patients being RELA fusion positive. Matsumoto et al. (11) found all CEs (5/5, 100%) had RELA fusions in their study. RELA fusion positive had been considered as a negative prognostic factor in ependymomas (16). However, Figarella-Branger et al. (18) and Lillard et al. (17) did not find that RELA fusion–positive STE had a poorer outcome; they hold GTR or near total resection (NTR) may overcome the deleterious effects of RELA fusion on survival (17–19). Our study showed nine patients with RELA fusion had favorable clinical outcomes, with no patient death at the last follow-up. We consider the favorable outcome in these patients may also owe to the high rate of GTR. The superficial locations of CEs have made GTR relatively straightforward, and 88.9% of RELA-positive patients underwent GTR in our series. Therefore, the impact of RELA fusion on survival of STE may need further supplement.

Based on our institutional case series and literature review, we found that CEs affected males more often than females. They were frequently involved in a single lobe, with frontal lobe being the most common, followed by the parietal lobe; the occipital lobe was the least common. Seizures (45.2%) were the commonest symptom in CEs, but only 15.3% of CEs were located in the temporal lobe. It was reported that only 30% lesional epilepsy series occurred in the extratemporal lobe (9). Hence, the phenomenon that CE’s lower incidence in temporal lobe and high association with epilepsy need vigilance. Furthermore, neurosurgeons should at least be reminded of the possibility of ependymoma when encountering the cortical mass presenting with seizures.

The incidence of anaplastic histology in CEs remained unclear. Several previous studies had indicated that most CEs (69.2–100%) were low grade (2, 5, 9). Van Gompel and colleagues (9) found that 75% of CEs (12/16 tumors) were WHO grade II. Likewise, Wang et al. (2) depicted 69.2% CEs (9/13 tumors) were WHO grade II in their study. Roncaroli et al. found that all CEs in their series were WHO grade II (5). However, higher rates of WHO grade III pathology in CEs were depicted in the studies by Liu et al. (20) (9/11 tumors, 81.8%) and Matsumoto et al. (11) (7/8 tumors, 87.5%). Khatri et al. (21) found that the incidence of WHO grade II CEs was equal with tumors with WHO grade III (50% vs. 50%). Our present study found that 50% (68/136 tumors) of CEs had WHO grade II pathology and equal number of grades 2 and 3 tumors at the time of first surgery.

The cortical location of CEs highlights the necessity of differentiating it from cortically located tumors such as pilocytic astrocytoma, ganglioglioma, and pleomorphic xanthoastrocytoma. Radiologically, our study exhibited CEs can be cystic (39.7%), solid (37.1%), and cystic-solid (23.2%), with low signal intensity on T1-weighted MRI and high signal intensity on T2-weighted MRI, and most of the CEs had enhancement on Gd-enhanced T1-weighted MRI, but the enhanced pattern varied. In solid and solid–cystic tumors, most of them were featured by homogeneous or heterogeneous enhancement. For cystic tumors, they were always characterized by large cyst with enhanced tumor nodule and cyst wall. Hence, the imaging features of CEs are non-specific, and preoperative radiologic diagnosis of CEs is quite difficult, and it can be easily misdiagnosed. Fortunately, different from the other glial tumors with diffuse infiltrating nature, CEs have a well-defined border on MRI, which may be a useful diagnostic point for the differential diagnosis.

Reported possible prognostic factors for supratentorial extraventricular ependymoma included patient age, tumor histology, extent of surgery, and adjuvant radiation (22–27). Our study found that tumor location, patient symptoms, WHO grade, extent of surgery, and irradiation were important and significant prognostic factors for PFS; WHO grade and extent of surgery were prognostic factors for OS in the univariate survival analysis. However, multivariate analysis indicated that only the extent of surgery and WHO tumor grade were prognostic factors for PFS or OS in CEs; GTR and WHO grade II were associated with improved survival. Safe maximal resection had long been considered as the optimum treatment for ependymomas. Besides, the superficial location and well-defined tumor border of CEs made GTR easy to perform. In the present series, 81.6% (111/136) of the tumors underwent GTR, and GTR was found to be a significant prognostic factor for longer PFS (*P* = 0.013) and OS (*P* = 0.003). Hence, our study supported that patients with CEs can also benefit from safe maximal tumor resection and re-resection may be appropriate for managing the initial STR with residual tumor.

The prognostic value of tumor WHO grade in ependymomas had been debated (28–33). Our study found that tumor pathology grade was a significant prognostic factor regarding PFS and OS in CEs; WHO grade II CEs had longer PFS and OS compared with WHO grade III tumors. In our study, only 11.9% (8/67 cases) of patients with WHO grade II tumors depicted subsequent recurrence, compared with 46% (29/63 cases) of patients with WHO grade III tumors (*P* < 0.001). In addition, 17.5% (11/63 cases) of patients with WHO grade III tumors died during the follow-up, which was significantly higher than patients with WHO grade II tumors (3.1%, *P* = 0.007). Hence, the fact that tumor grade influences the PFS and OS in patients with CEs, independently of other factors, should be considered in the design of future clinical treatment guidelines.

Controversial opinions had been depicted in the literature regarding the efficacy of radiotherapy for the treatment of STEs (34–36). Recent studies suggested that radiotherapy should be applied in patients with WHO grade III tumors or in cases in which STR had been achieved in STEs (13, 26, 37). However, no previous study had therefore investigated the value of radiotherapy in CEs. In the present study, 69.1% (47/68 cases) of the patients with WHO grade III CEs received postoperative irradiation, and they depicted significantly longer OS compared with those without irradiation (*P* = 0.008). However, the utilization of postoperative irradiation did not get prolonged OS (*P* = 0.371) in WHO grade II CEs. Thus, our findings suggested the beneficial role of adjuvant RT for WHO grade III CEs, but the impact of RT on tumor control in WHO grade II CEs should be interpreted with caution.

To the best of our knowledge, our study represents the largest case series that focused on the CEs in the modern neurosurgical era. The present assessments confirmed that GTR is the optimum treatment of choice in CEs. In addition, tumor pathology is a significant prognostic factor regarding PFS and OS in CEs. We recommend that postoperative adjuvant radiotherapy is necessary in WHO grade III CEs, but the application of irradiation in WHO grade II CEs should be treated with caution.

### Limitations

Several limitations should be reminded in our study. First, because of the rarity of CEs, it was difficult to conduct a survival analysis based on an institutional data. Thus, to maximize our sample size and generate statistical significance, we included patients from literature review, and some intrinsic limitations still need attention. Second, only ependymoma-afflicted patients hospitalized after 2017 in our series had been screened for C11orf95-RELA fusion, and YAP1 fusions analysis was not included in the present series; further prospective multicenter studies are needed to investigate the incidence of C11orf95-RELA and YAP 1 fusion in CEs. Lastly, despite the present study representing the largest series of CE patients to date, our sample size was still relatively small, and further studies with larger sample sizes are needed.

## Conclusion

In summary, CEs are rare and account for 3.5 to 5.7% of ependymomas. CEs most commonly occur in the frontal lobe, presenting with seizures. WHO grade III tumors constituted 50% of the CEs, which was identical with the proportion of WHO grade II tumors. CEs may have a higher rate of RELA fusions, but generally have favorable prognosis. The extent of surgical resection and WHO tumor grade were significant prognostic factors. GTR with close follow-up was the optimum treatment, and WHO grade II CEs had significantly longer PFS and OS. The utilization of postoperative irradiation can significantly prolong the OS in WHO grade III CEs, but not in the WHO grade II tumors.

## Data Availability Statement

The datasets generated for this study are available on request to the corresponding author.

## Ethics Statement

The studies involving human participants were reviewed and approved by the Ethics Committee of West China Hospital. Informed consent was obtained from all individual participants included in the study.

## Author Contributions

QW and XH conceived the study design. QW, JC, and JL were responsible for patient recruitment and literature review. QW, JC, SZ, and WL were responsible for statistical analysis. QW, YJ, and XH drafted the article. All authors discussed the results, revised the draft, and approved the final manuscript.

## Conflict of Interest

The authors declare that the research was conducted in the absence of any commercial or financial relationships that could be construed as a potential conflict of interest.
